# Retraction and correction of scientific articles due to conflicts of interest: a cross‑sectional analysis of the Retraction Watch database

**DOI:** 10.1186/s41073-026-00236-9

**Published:** 2026-07-08

**Authors:** Ružica Bočina, Antonija Mijatović, Ana Marušić

**Affiliations:** 1 Department of Pathology, Forensic Medicine & Cytology, University Hospital of Split, Split, Croatia; 2 Department of Research in Biomedicine and Health, Centre for Evidence-Based Medicine, School of Medicine, University of Split, Split, Croatia

**Keywords:** Conflict of Interest, Retraction of Publication, Editorial Policies, Publishing/ethics, Scientific Misconduct, Databases, Bibliographic, Retraction Watch database, Research Integrity

## Abstract

**Background:**

Conflicts of interest (CoI) can undermine trust in scientific publishing, yet empirical evidence on how often CoI lead to editorial action and how journals respond remains limited. This study aimed to characterise the frequency, patterns, and editorial handling of CoI‑related notices in the scholarly literature using an openly available, multidisciplinary dataset.

**Methods:**

We conducted a descriptive cross‑sectional analysis of publicly available records from the Retraction Watch (RW) database, accessed via Crossref, from inception to 23 April 2026. All editorial notices in which CoI was explicitly mentioned as the sole or one of several stated reasons were included, encompassing retractions, corrections, expressions of concern, and reinstatements. For each record, we extracted article and notice dates, article type, scientific field, country of the corresponding author, number of authors and institutions, publisher, and all stated reasons for the notice. Data were analysed using descriptive statistics and bootstrap‑based confidence intervals.

**Results:**

We identified 886 CoI‑related records (748 retractions, 39 corrections, 97 expressions of concern, and 2 reinstatements). CoI was the sole stated reason in 95 cases (10.7%), including 32 retractions, 11 corrections, and 52 expressions of concern; in the remaining notices, CoI most commonly co‑occurred with concerns such as compromised peer review, authorship or affiliation problems, journal or institutional investigations, and broader ethical violations. The median time from publication to editorial action was longest for expressions of concern and shortest for corrections, with wide variability across records and notice types. CoI‑related notices were concentrated in basic life, biomedical, and health sciences, and were unevenly distributed across high‑output countries and a small number of large publishers, particularly open‑access outlets. Most affected articles were original research papers with multi‑author, multi‑institution teams. Among articles with determinable author gender, both first and last authors were more often male than female, although these patterns were exploratory and lacked a baseline comparator.

**Conclusions:**

Editorial actions explicitly linked to CoI remain relatively uncommon compared with the broader volume of retractions and corrections, and CoI are rarely cited in isolation. The frequent co‑occurrence of CoI with other forms of misconduct or procedural problems suggests that undisclosed or poorly managed CoI often signal deeper weaknesses in disclosure practices, editorial oversight, and research governance rather than isolated administrative lapses. Strengthening CoI policies, transparency, verification mechanisms, and linked metadata systems may help detect problems earlier and support more consistent, graduated editorial responses.

## Introduction

Research integrity is the cornerstone of the academic and scientific community, aimed at preserving honesty, credibility, and responsibility at all stages of the research process, from the formation of research questions to the publication and interpretation of results. Its purpose is to ensure that scientific knowledge remains credible, verifiable, and socially useful. Without integrity, science loses its ability to address crucial societal challenges and safeguard public trust [1]. A pivotal element of research integrity is transparency, which requires open disclosure of all aspects of research, including funding sources, potential conflicts of interest (CoI), methodological procedures, and limitations. Transparent reporting allows for the successful replication of findings, reliability checks for data, and the identification of possible sources of bias [2]. The commitment to maintain research integrity does not rest solely on researchers but is distributed across all participants in the scientific system: reviewers, editors, and publishers alike. International organizations such as the Committee on Publication Ethics (COPE), the International Committee of Medical Journal Editors (ICMJE), and the World Association of Medical Editors (WAME) have established clear guidelines on ethical publishing, emphasizing the importance of disclosing CoI, proper citation, and editorial impartiality [3–5].

The frequency of article retractions has increased over recent decades, a trend that likely reflects both heightened detection and correction of flawed research and a growing willingness to withdraw articles whose credibility is in doubt [6, 7]. Research misconduct, such as plagiarism, data fabrication, and fraudulent peer review, remains the most common cause of retraction, but CoI have become increasingly recognized as a significant and complex risk [6, 8]. In scientific publishing, CoI can be defined as circumstances in which secondary interests, including financial gain, professional ambition, or personal relationships, risk compromising the objectivity of research and publishing decisions [7, 9]. Financial conflicts, especially in the biomedical field, typically include undisclosed industry funding, consulting relationships, or shares in a company, while non‑financial conflicts may involve a drive for academic promotion, reputational gain, or deeply held beliefs [9, 10]. Both financial and non‑financial conflicts pose threats to the integrity of the evidence base, as they can distort findings, influence the presentation of results, and undermine trust in science [10, 11]. Reviews and empirical studies show that CoI is frequently underreported in manuscripts, and even when required, many authors and editors fail to fully disclose relevant interests, leading to hidden biases and compromised transparency. Therefore, detecting and addressing CoI is essential to protect scientific credibility and advance reliable, socially valuable research [10, 12, 13].

Efforts to improve the transparency of CoI disclosure have led to the development of international policies and standardized reporting tools, most notably the ICMJE uniform disclosure form, first introduced in 2009 and updated in February 2021 — replacing the term "conflicts of interest" with the broader concept of "disclosure of interests" and expanding the declared categories to reduce negative connotations and promote more comprehensive transparency [4, 14]. COPE further recommends that all participants in the publication process (authors, editors, and reviewers) have clear, publicly available CoI policies and that journals ensure regular updates and consistent management of these disclosures [3]. Despite these frameworks, compliance and practical enforcement remain inconsistent, allowing underreporting, vague definitions, and administrative differences to undermine the goal of universal transparency and can unintentionally permit CoI to influence the scientific record. Surveys of environmental, occupational, and public health journals show that many journals’ CoI policies do not explicitly apply to editors or reviewers, and that formal enforcement mechanisms are rare, underscoring substantial variability in policy content despite the availability of international guidance [15].

Large‑scale analyses of retraction patterns also reveal that, beyond personal misconduct, systemic weaknesses in editorial processes and inconsistent CoI management significantly contribute to the persistence of compromised publications [13]. Recently, the Retraction Watch (RW) project has become a central tool for monitoring such issues: initially launched in 2010 as a blog by science journalists Ivan Oransky and Adam Marcus to track retractions as a "window into the scientific process," it later expanded to include a curated database that systematically records retracted articles and selectively includes related corrections and expressions of concern across disciplines when these are identified by RW staff or readers [16, 17]. In September 2023, Crossref, a non‑profit membership organization providing open, community‑owned infrastructure for scholarly metadata and persistent identifiers, acquired the RW Database and made its contents openly available via its application programming interfaces (APIs) and metadata services. This integration substantially increases the coverage, accessibility, and interoperability of retraction information by combining publisher‑deposited metadata with RW curated records, thereby enabling more complete and reliable detection of problematic articles throughout the scholarly ecosystem [17, 18]. Current evidence highlights that many retracted articles arise in settings where editorial safeguards and retraction procedures are incompletely implemented, so ethically problematic studies may remain available or insufficiently labelled for prolonged periods, allowing them to continue influencing clinical practice and subsequent research [19, 20]. Large‑scale descriptive and bibliometric analyses across the scientific literature show that ethical violations and other forms of research misconduct remain leading reasons for retraction, with marked geographic, disciplinary, and journal‑level differences that underscore the need for clearer publisher responsibilities and harmonized international standards. These studies also document substantial variation in retraction practices between journals and regions, as well as a complex interaction between author‑level characteristics and editorial decisions, reinforcing the importance of a robust, cross‑disciplinary approach to safeguarding research integrity across specialties and publication models, including open‑access outlets [20, 21].

These developments collectively emphasize the urgent need to strengthen journal practices, promote rapid and transparent reporting, and leverage open infrastructures such as the RWCrossref collaboration to mitigate the risks posed by CoI and enhance the trustworthiness of the scientific literature for the global community [17–19]. Alongside the RW–Crossref infrastructure, several other tools and databases have been developed to track retracted and problematic research. These include Argos (Scitility PBC), a science-integrity platform launched in 2024 that assigns risk scores to articles by analyzing retraction cascades, author publication histories, and citation patterns; retractBASE, an open database developed by the Spanish National Research Council (CSIC) and the University of Córdoba that compiles retracted publications and withdrawals from 2000 onward using Crossref and OpenAlex as source infrastructure; and Crossmark, a Crossref service that embeds a real-time status indicator in published articles and their PDFs, alerting readers to corrections, retractions, or updates registered by the publisher [22–27]. Together, these complementary resources illustrate the growing ecosystem of tools aimed at improving the transparency and discoverability of editorial interventions across the scholarly literature.

Against this background, this study was guided by three research questions: How frequently do CoI appear as the sole versus co-occurring reason across different types of editorial notices (retractions, corrections, and expressions of concern), and with which other forms of misconduct do they most commonly co-occur? What are the temporal, geographic, disciplinary, and publisher-level characteristics of articles subject to CoI-related editorial action? Are there differences in authorship patterns (including team size, number of institutional affiliations, and gender distribution) between articles that were retracted, corrected, or flagged with expressions of concern due to CoI? By addressing these questions, this study aims to provide empirical evidence to inform journal policies, editorial decision‑making, and the design of international systems for detecting, labelling, and managing CoI‑related editorial actions.

## Methods

### Study design and data source

This descriptive cross‑sectional study used publicly available data from the Retraction Watch (RW) database to characterize the frequency, patterns, and editorial handling of notices citing conflicts of interest (CoI). The RW database primarily focuses on indexing retractions across scientific disciplines and, in addition, includes a subset of related editorial notices such as corrections and expressions of concern when these are identified by RW staff, reported by readers, or linked to retracted articles [16]. Since September 2023, the RW database has been openly accessible via Crossref, which integrates RW‑curated records with publisher‑deposited metadata, thereby improving the coverage and accessibility of retraction information through a common infrastructure [17, 18].

All records indexed in the RW database from its inception through 23 April 2026 were screened, and those in which CoI was explicitly mentioned as the sole or one of several stated reasons for an editorial notice were included. Notices were eligible regardless of whether CoI was cited independently or alongside other reasons. All types of editorial notices (retractions, corrections, expressions of concern, and reinstatements) were examined to capture the full spectrum of editorial interventions associated with CoI. In total, 886 CoI‑related records met these criteria and formed the main analytic sample.

Within this overall sample, we additionally identified a subset of articles for which an initial correction or expression of concern citing CoI was later followed by a retraction. For this escalation subset, we recorded whether a retraction was ultimately issued and the dates of both the first CoI‑related notice and the subsequent retraction, in order to calculate the time from initial notice to retraction.

### Data collection and variables

The RW retractions dataset (CSV) was downloaded from the Crossref GitLab repository on 23 April 2026 [28]. Because the RW database is continuously updated, all analyses in this manuscript were restricted to records available up to 23 April 2026 in order to provide a complete and reproducible snapshot of CoI‑related editorial notices at that point in time. Corrections and expressions of concern are only included in the RW database when they are identified by RW staff, reported by readers, or linked to articles that have attracted editorial attention; therefore, the counts of these notice types in our dataset should not be interpreted as a comprehensive census of all corrections and expressions of concern in the scholarly literature.

Data collection and organization were performed in a spreadsheet, using Microsoft Excel for Microsoft 365 [29]. For each included article, the following variables were extracted: type of notice issued (retraction, correction, expression of concern, reinstatement), whether CoI was the sole or one of multiple stated reasons, any additional reasons cited, the original article’s publication date, the date of the retraction or editorial notice, the country of the corresponding author’s institution, article type (e.g. original research, review, clinical trial), scientific field or category, number of authors, number of affiliated institutions represented, and the journal publisher. To ensure data integrity and accuracy, every editorial record was individually cross‑checked against the information provided on the official website of the original journal or publisher. For the analysis of reasons for editorial intervention, all reason codes assigned in the RW database were extracted. Following the RW Database User Guide (Appendix B), categories that describe characteristics of notices or editorial handling procedures rather than underlying causes (for example "Date of Article and/or Notice Unknown", "Updated to Retraction", "Upgrade/Update of Prior Notice(s)", "Removed", and "Temporary Removal") were treated as descriptive metadata [30]. These handling‑related categories were excluded from the analysis of reasons for retraction, correction, and expression of concern, and only categories representing substantive concerns about the article (such as plagiarism, falsified or unreliable data, fake or compromised peer review, authorship or affiliation issues, and ethical violations) were retained. Notices classified as reinstatements were recorded descriptively but excluded from stratified analyses because only two such cases were identified in the dataset, resulting in an insufficient sample for meaningful quantitative evaluation.

To assess gender representation among first and last authors, we first determined gender manually whenever this could be reliably inferred from the author’s full name and publicly available information (e.g. institutional webpages, professional profiles, or other publications), after retrieving full given names for records in which only initials or incomplete first names were available by checking the original articles via their Digital Object Identifiers (DOIs) and journal websites. For the remaining authors whose gender could not be confidently established by manual verification, we used the web‑based tool Genderize.io, which predicts an individual’s gender from their first name and provides an associated probability score [31]. We assigned gender only when the predicted probability was at or above a pre‑specified threshold of 60%; names with low‑probability or ambiguous predictions were coded as "gender undetermined" and excluded from gender‑stratified analyses. Gender analyses were conducted separately for retracted, corrected, and flagged articles to explore potential gender‑related patterns within author teams affected by CoI‑related editorial interventions.

### Statistical analysis

Statistical analysis was performed using Python version 3.11. [32]. To estimate the median and corresponding 95% confidence intervals (CI) for variables such as the time from publication to editorial notice, the time from initial CoI‑related notice to retraction, the number of authors per article, and the number of institutional affiliations, the bootstrap resampling method (10,000 iterations) was employed. This non‑parametric approach was selected for its robustness in estimating parameters from skewed distributions and small to intermediate sample sizes. Data management was carried out using the pandas library, and numerical computation and generation of bootstrap samples were performed with NumPy [33, 34]. All analyses were conducted separately for articles that were retracted, corrected, or received expressions of concern due to issues with CoI.

## Results

### Overview of included records

A total of 886 records related to conflict of interest (CoI) were identified in the RW database. Among these, 748 were retractions, 39 were corrections, 97 were expressions of concern, and 2 were reinstatements (Fig. 1). CoI was the sole stated reason in 95 cases overall (10.7% of all records), including 32 retractions, 11 corrections, and 52 expressions of concern.

**Fig. 1:**
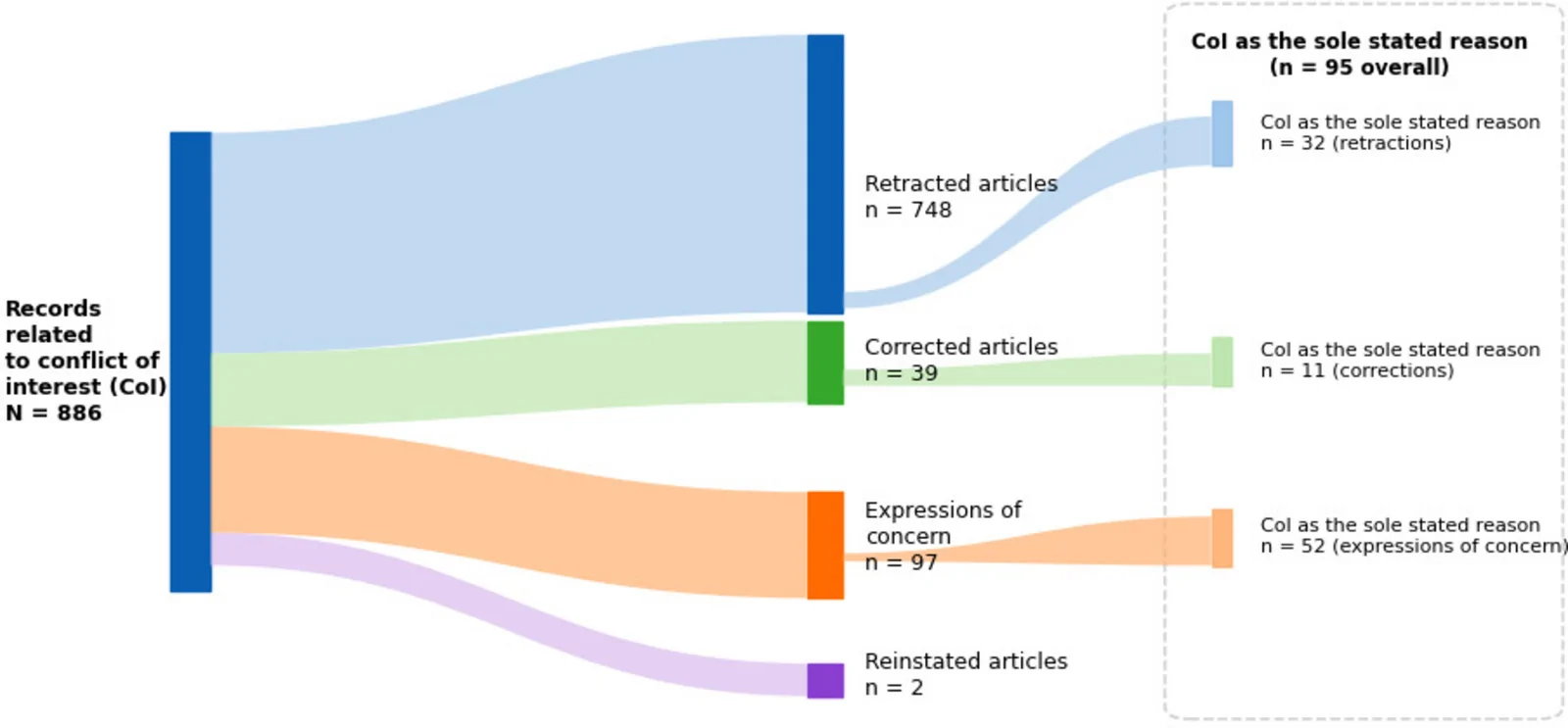
Overview of included articles and editorial outcomes (Sankey diagram created in Python). CoI—conflict of interest

In most records, CoI was reported alongside additional concerns. Overall, 98 distinct RW "reason" categories were recorded across the included notices. In line with the RW Database User Guide, categories describing notice characteristics or editorial handling (such as "Date of Article and/or Notice Unknown", "Updated to Retraction", "Upgrade/Update of Prior Notice(s)", or "Removed") were treated as descriptive metadata and excluded from the substantive analysis of reasons. The remaining 39 categories captured underlying causes for editorial intervention, with the most frequent including plagiarism, falsified or unreliable data, fake or compromised peer review, authorship or affiliation issues, journal or institutional investigations, concerns regarding research ethics, and author objections, as summarised in Table 1.

**Table 1:** Substantive reasons for which scientific articles were retracted, corrected, flagged as expressions of concern, or reinstated, together with the number and percentage of records in which each reason was identified after re‑coding Retraction Watch database "Reasons" into underlying causes for editorial intervention*

Reason	No. of Articles (Percentage)
Peer Review Concerns	493 (55.6)
Investigation by Journal/Publisher	403 (45.5)
Authorship/Affiliation Concerns	280 (31.6)
Results/Conclusions Concerns	250 (28.2)
Objections by Author(s)	216 (24.4)
Third-Party Concerns	156 (17.6)
Concerns/Issues about Referencing/Attributions	142 (16.0)
Rogue Editor	118 (13.3)
Data Concerns	100 (11.3)
Article Errors	66 (7.4)
Breach of Policy by Author	52 (5.9)
Human Subjects/Ethics Approval	51 (5.8)
Image Concerns; Plagiarism/Duplication	35 (4.0)
Institutional/Corporate Issues	30 (3.4)
Bias Issues or Lack of Balance	14 (1.6)
Author Unresponsive	13 (1.5)
Ethical Violations by Author; Misconduct—Official Investigation(s) and/or Finding(s)	12 (1.4)
Concerns/Issues about Article	11 (1.2)
Lack of Approval from Author	9 (1.0)
Concerns/Issues about Methods; Copyright Claims	7 (0.8)
Legal Reasons/Legal Threats	4 (0.5)
Cites Retracted Work; Misconduct by Author	3 (0.3)
Criminal Proceedings; Miscommunication by Author; Misconduct—Official Investigation/Finding; Taken from Dissertation/Thesis	2 (0.2)
Civil Proceedings; Complaints about Author; Concerns/Issues about Animal Welfare; Doing the Right Thing; Error by Journal/Publisher; Miscommunication by Journal/Publisher; Paper Mill; Temporary Removal	1 (0.1)

*Reasons were derived from the Retraction Watch database "Reasons" field, with categories describing notice metadata or editorial handling (e.g. "Updated to Retraction", "Removed") excluded in accordance with the Retraction Watch Database User Guide (Appendix B), and only categories representing substantive concerns about the article retained. Percentages were calculated based on the total number of analyzed articles (*N* = 886). As a single retraction notice may report more than one reason, percentages do not add up to 100%

Of the 1772 first and last authors across all 886 included articles, gender was determined manually for 1116 (63%); for the remaining authors, gender was inferred using Genderize.io with a probability threshold of ≥ 60% for 637 (36%), while gender could not be determined for 19 (1%).

### Retractions

Out of 748 retracted articles, CoI was cited as the sole reason for retraction in 32 cases (4.3%). The most common co-occurring reasons included peer review concerns (*n* = 429), journal or publisher investigations (*n* = 379; 50.7%), authorship or institutional affiliation issues (*n* = 278), and concerns about results/conclusions (*n* = 231). The median time from publication to retraction was 948 days (95% CI = 825–1052 days). Most retracted articles were published between 2012 and 2021, with publication dates ranging from June 1977 to October 2025.

The leading countries of origin were China (*n* = 284), Pakistan (*n* = 185), India (*n* = 150), Saudi Arabia (*n* = 144), and the United States (*n* = 109). Regarding publishers, the largest numbers of retracted articles were published by Elsevier (*n* = 195, 26%), Public Library of Science (PLOS) (*n* = 180, 24%), Frontiers (*n* = 134, 17.9%), Wiley (*n* = 42, 5.6%), Springer Nature (*n* = 27, 3.6%). Most articles were original research papers (*n* = 584), with review articles (*n* = 71) and clinical studies (*n* = 46) also represented. The predominant scientific fields were basic life sciences (*n* = 365), biomedical and health sciences (*n* = 230), business/technology (*n* = 192), and physical sciences (*n* = 184). The median number of authors per retracted article was 5 (95% CI = 5–6), with an interquartile range of 3–8 authors, and the median number of affiliated institutions per retracted article was 3 (95% CI = 3–4), with an interquartile range of 2–5 institutions.

### Corrections

Among the 39 corrections, CoI was the sole reason for correction in 11 cases (28.2%). The most common additional reasons included article errors (*n* = 17), involvement of third parties (*n* = 10), institutional or corporate issues (*n* = 4), and concerns on results/conclusions (*n* = 3). The median time from publication to correction was 669 days (95% CI = 119–774 days), with most corrected articles published between 2007 and 2017. Publication dates ranged from February 2002 to February 2023.

The United States was the leading country of origin (*n* = 29), followed by the United Kingdom (*n* = 14), Canada (*n* = 6), and France and Italy (*n* = 5 each). Publishers with the largest numbers of corrected articles were the American Heart Association (*n* = 7), the Italian Society of Medical Statistics and Clinical Epidemiology (SISMEC), the Massachusetts Medical Society, and Oxford Academic (*n* = 3 each). Most corrected articles were original research (*n* = 20), with review articles (*n* = 9) and clinical studies (*n* = 7) also present. Health sciences (*n* = 31), basic life sciences (*n* = 19), and environmental sciences (*n* = 3) were the most represented scientific fields. The median number of authors per corrected article was 6 (95% CI = 4–10), with an interquartile range of 4–15 authors, and the median number of affiliated institutions per corrected article was 4 (95% CI = 3–5), with an interquartile range of 2–6 institutions.

For the 39 corrected articles, the gender of the first author could not be determined in 1 case. Among the 38 articles for which first-author gender could be determined, 9 (23.7%) first authors were female. The gender of the last author could not be determined in 2 of the 39 articles. Among the remaining 37 articles, 10 (27%) had a female last author. 

Over time, 7 out of 39 corrected articles were retracted. The median time from correction to retraction was 1107 days (95% CI = 1104–1632 days).

### Expressions of concern

Among the 97 articles flagged with expressions of concern, CoI was the sole listed reason in 52 cases (53.6%). Other commonly cited reasons included data-related concerns (*n* = 26), editorial investigations (*n* = 24), updates to retraction notices (*n* = 18), and concerns about results/conclusions (*n* = 16).

The median time from publication to expression of concern was 2444 days (95% CI = 2102–2725 days). Publication dates ranged from April 2007 to December 2023.

Most articles in this group originated from Japan (*n* = 53), the United States (*n* = 34), and the United Kingdom (*n* = 10), with smaller contributions from Italy (*n* = 6). The predominant article type was original research (*n* = 82), and most articles belonged to the health sciences (*n* = 75) and basic life sciences (*n* = 68). The largest numbers of affected articles were published by PLOS (n = 54), Taylor & Francis (*n* = 25), Hindawi (*n* = 5), Verduci Editore (*n* = 4), and SAGE Publications (*n* = 3). The median number of authors per article flagged with an expression of concern was 7 (95% CI = 5–8), with an interquartile range of 5–11 authors, and the median number of affiliated institutions was 3 (95% CI = 3–4), with an interquartile range of 2–5 institutions.

For the 97 articles, 17 (17.5%) first authors were female. The gender of the last author could not be determined in 2 of the 97 articles. Among the remaining 95 articles, 12 (12.6%) had a female last author.

Out of the 97 articles tagged with an expression of concern, 22 (22.7%) were subsequently retracted. The median time from expression of concern to retraction was 237 days (95% CI = 237–237 days).

All three notice types were relatively uncommon between 1999 and approximately 2014, with counts remaining consistently low (Fig. 2). Retractions increased substantially after 2015 and accelerated further after 2021, reaching a peak in 2025. Corrections remained comparatively infrequent throughout the study period. Expressions of concern were infrequent before 2020 but increased thereafter, peaking in 2023.

**Fig. 2:**
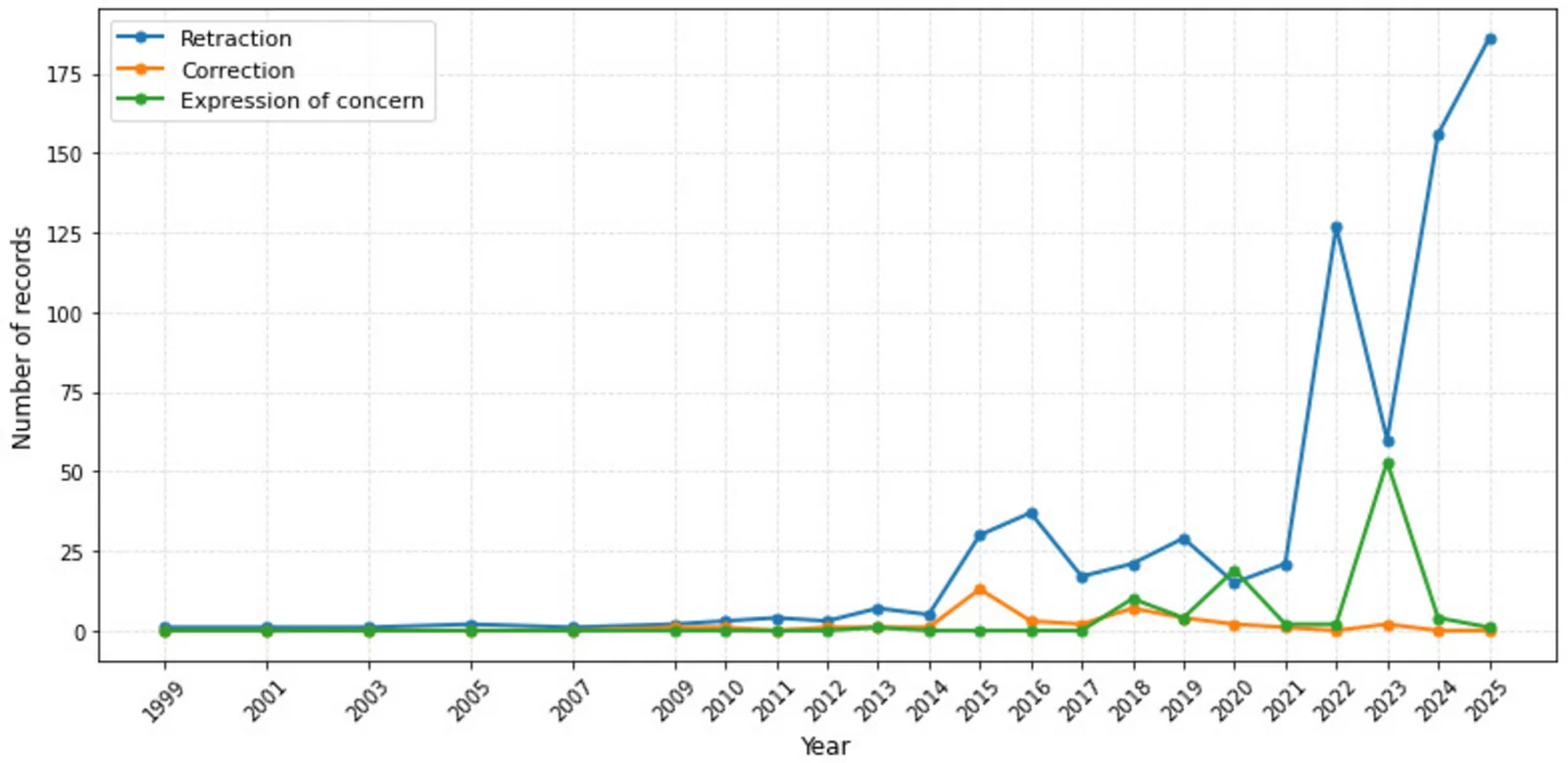
Annual number of CoI-related editorial notices by notice type (retractions, corrections, and expressions of concern) in the RW-Crossref database from 1999 to 2025. Records from 2026 were excluded because data collection ended on 23 April 2026

## Discussion

To our knowledge, this is the first study to characterize conflicts of interest as explicitly stated reasons for retractions, corrections, and expressions of concern in a large, multidisciplinary dataset derived from the openly available RW–Crossref database. By analyzing 886 CoI‑related editorial notices, we provide empirical benchmarks for how often CoI is cited alone versus in combination with other problems across different types of editorial actions: CoI was the sole stated reason in approximately 4.3% of retractions, 28.2% of corrections, and 53.6% of expressions of concern. These proportions reveal a clear gradient in editorial response: CoI alone is rarely deemed sufficient to warrant retraction, whereas it more frequently constitutes the primary driver of corrections and expressions of concern. These findings extend previous work on retraction patterns by focusing specifically on how CoI features in formal editorial decisions, rather than treating it only as a background characteristic of misconduct cases [20, 35].

The low frequency of CoI as the sole retraction trigger reflects the conceptual framework established by COPE, which recommends that undisclosed CoI typically warrant correction or a published erratum rather than outright retraction, unless the CoI is deemed to have fundamentally compromised the validity of the work [3]. This distinction between administrative responses appears to be functioning in practice, consistent with the low proportion of retractions in which CoI is cited as the only problem in our dataset. However, when CoI does appear alongside other reasons in retracted articles, it overwhelmingly co‑occurs with serious research misconduct, as detailed in the Results. This syndromic presentation suggests that CoI operates not as an isolated breach but as part of a broader ecosystem of systemic failures in manuscript review, data integrity, and institutional oversight [35].

In our dataset, CoI‑related retractions were typically issued within a few years of publication, whereas corrections appeared after a broadly similar or slightly shorter delay, and expressions of concern often emerged only many years later. This pattern suggests that once problems are considered severe enough to justify retraction, editorial action tends to be comparatively more prompt, while CoI‑related concerns that result in expressions of concern may linger in the literature for prolonged periods before they are formally acknowledged. Such delays are concerning, because previous studies have shown that retracted biomedical articles frequently continue to be cited long after retraction, that only a small minority of post‑retraction citations explicitly acknowledge the article’s retracted status, and that retraction alone is an inadequate measure for preventing the use of invalid findings in subsequent research and clinical practice [36, 37].

The geographic distribution of CoI-related editorial notices in our dataset reflects absolute counts and should not be interpreted as evidence of differential misconduct rates across countries. CoI-related retractions were numerically concentrated in China (*n* = 284), Pakistan (*n* = 185), India (*n* = 150), Saudi Arabia (*n* = 144), and the United States (*n* = 109) — five countries that together accounted for the large majority of retracted articles in our dataset, while the United States simultaneously dominated corrections (*n* = 29), suggesting that different types of editorial responses may be distributed unevenly across research systems even within the same high-output countries. Expressions of concern originated predominantly from Japan (*n* = 53), the United States (*n* = 34), and the United Kingdom (*n* = 10).

These patterns are consistent with prior bibliometric analyses documenting that absolute retraction counts tend to cluster in high-output research systems such as China and the United States [20, 21, 38]. Taken together with our own data, this suggests that country-level differences in CoI-related retractions, corrections, and expressions of concern may reflect not only underlying research volume but also variation in editorial infrastructure and willingness to formally label CoI-related problems; however, the present study was not designed to disentangle these mechanisms and cannot establish their relative contribution.

At the publisher level, CoI-related retractions in our dataset were concentrated in a small number of large, predominantly open-access publishers, particularly Elsevier (*n* = 195), PLOS (*n* = 180), and Frontiers (*n* = 134), with Wiley (*n* = 42) and Springer Nature (*n* = 27) contributing smaller but still notable numbers. Corrections and expressions of concern were likewise clustered, with corrections most frequently issued by the American Heart Association and a small group of major society and commercial publishers, and expressions of concern primarily originating from PLOS, Taylor & Francis, Hindawi, and SAGE. These distributions cannot be interpreted as evidence that CoI problems are intrinsically more prevalent in journals from these publishers; instead, they likely reflect differences in publication volume, editorial policies for labelling CoI in notices, and the extent to which publishers participate in infrastructures such as the RW–Crossref database, which facilitate the registration and discoverability of editorial interventions. Prior work has similarly shown that retractions tend to cluster in a subset of high-output journals and publishers, and that cross‑journal and cross‑country differences in retraction patterns must be interpreted in light of variation in publication volume, indexing practices, and the way databases and journals label retracted articles, rather than as direct proxies for misconduct prevalence [38, 39].

Our finding that more than half of expressions of concern in our sample cited CoI as the sole stated reason (53.6%) suggests that editors may use this notice type as a cautious, intermediate response to prominent undisclosed or disputed conflicts, particularly in high‑stakes biomedical fields. In this context, expressions of concern allow journals to alert readers to potential CoI‑related problems while investigations are ongoing, without immediately implying that the article’s findings are invalid or warrant retraction [40]. However, only about one in five CoI‑related expressions of concern in our dataset (22.7%) were followed by a subsequent retraction, and we lacked information on whether the remaining notices ultimately led to corrections, informal resolutions, or no further action.

This pattern indicates that many CoI‑related concerns flagged through expressions of concern may remain unresolved from the perspective of readers and bibliographic databases, and underscores the need for clearer reporting on the outcomes and duration of editorial investigations in future work.

### Strengths and limitations

This study has several strengths. It uses the largest openly accessible infrastructure currently available for tracking retractions and related editorial actions, drawing on the Retraction Watch–Crossref collaboration to examine CoI-related notices across multiple disciplines, countries, publishers, and notice types within a single analytic framework. It also applied manual verification of journal notices and article metadata, and distinguished whether CoI was cited as the sole stated reason or alongside other concerns, which enabled a more granular description of how CoI is handled in retractions, corrections, and expressions of concern.

However, several limitations should be acknowledged. First, the RW database primarily focuses on retractions and includes only a subset of corrections and expressions of concern, which are entered when identified by RW staff, reported by readers, or linked to already flagged articles; accordingly, the counts of corrections and expressions of concern in this study should not be interpreted as a comprehensive census of such notices across the scholarly literature. A further limitation relates to how CoI is represented in the RW database and in notice texts. The database records the presence of CoI concerns, but generally does not provide sufficient detail to distinguish between financial and non‑financial conflicts of interest, nor between intentional concealment and negligent or inadvertent disclosure failures. Consequently, all CoI‑related notices in this study were analysed as a single category, and the findings cannot speak to the relative contribution of different types of conflicts. Future studies based on full‑text examination of retraction notices, editorial statements, and institutional investigation reports may be able to further characterise the nature of the underlying conflicts and their relationship to editorial outcomes.

Third, our geographic and publisher-level analyses are based on absolute counts and were not standardised by underlying publication volume, disciplinary composition, journal output, or national research production. These comparisons should therefore be interpreted descriptively and not as direct measures of misconduct prevalence or editorial quality. Fourth, although author-gender assessment was strengthened through manual retrieval of full given names and conservative use of Genderize.io only for unresolved cases, gender assignment remained probabilistic for a subset of authors and should be interpreted with caution, especially because the analysis was exploratory and lacked a baseline comparator from the broader published literature or from retractions due to other causes. Fifth, the statistical analyses were intentionally descriptive. Bootstrap confidence intervals were used to summarise skewed distributions, but the study did not include vertical comparisons with all retractions or horizontal comparisons with retractions due to other specific reasons, so temporal and authorship patterns should not be overinterpreted as evidence of distinct CoI-specific mechanisms.

Finally, because this was an observational cross-sectional study based on secondary notice texts, it could not determine whether undisclosed CoI existed but remained undetected at submission, emerged later, or was only formally recognised after other concerns had arisen. Nor could it establish the final outcome of many expressions of concern that did not progress to retraction.

### Implications and future directions

Despite these limitations, the findings have several practical implications. First, the rarity of CoI as the sole stated reason for retraction, together with its much greater prominence in corrections and expressions of concern, supports a more graduated view of editorial responses in which undisclosed CoI often functions as a warning signal that may precede, accompany, or amplify broader concerns about research integrity rather than acting as an isolated trigger. This underscores the importance of making CoI disclosures more structured, complete, and verifiable before publication, rather than relying primarily on post-publication editorial action.

Second, the concentration of CoI-related notices within particular countries, publishers, and biomedical fields suggests that future work should move beyond absolute counts and incorporate denominator-based analyses, such as publication volume, field-normalised output, and journal-level exposure measures, in order to distinguish true differences in risk from differences in visibility, labelling, and editorial infrastructure. Third, future studies should prioritise primary-source coding of notice texts and, where possible, the underlying articles and disclosure forms, so that financial and non-financial CoI, as well as intentional versus negligent non-disclosure, can be analysed separately. This would substantially improve the policy relevance of the literature, because these forms of CoI likely require different preventive and corrective strategies.

Fourth, more robust comparative work is needed on the timeliness and consequences of CoI-related editorial actions. This includes comparing CoI-related retractions with retractions due to other reasons, tracking what happens to expressions of concern that do not culminate in retraction, and examining whether corrections adequately mitigate the downstream citation and use of affected articles. At a systems level, the findings support continued development of interoperable, machine-readable infrastructures linking disclosure statements, editorial notices, and bibliographic metadata, so that readers, journals, institutions, and indexing services can detect and communicate CoI-related concerns more consistently.

## Conclusions

In this cross-sectional analysis of the RW database, conflicts of interest were explicitly cited in a modest number of editorial notices and were rarely listed as the sole reason for retraction; instead, they most often appeared alongside other indicators of editorial, procedural, or research-integrity problems. CoI as a sole stated reason was substantially more common in corrections and especially in expressions of concern than in retractions, suggesting that journals often treat undisclosed CoI as an issue warranting graduated editorial response unless the integrity of the work is considered fundamentally compromised.

Taken together, these findings suggest that CoI-related notices should not be understood simply as isolated administrative failures. Rather, they may mark broader weaknesses in disclosure practice, editorial oversight, and the systems used to detect, label, and communicate concerns about the published record. Strengthening disclosure policies, improving verification and metadata standards, and developing more transparent follow-up pathways for corrections and expressions of concern may help identify problems earlier and support more consistent protection of the scientific record.

## Data Availability

The dataset used and analyzed in the study was obtained from the publicly available Retraction Watch Database in collaboration with Crossref, which provides a downloadable data file of retraction metadata for research use. The specific data sheet and accompanying documentation can be accessed free of charge from the Retraction Watch/Crossref data resource website. All the data from this study are included in the article and its supplementary document, publicly available on Zenodo: (https://zenodo.org/records/20478570).

## Abbreviations

APIs: Application Programming Interfaces
BMJ: British Medical Journal
CI: Confidence Interval
CoI: Conflict(s) of Interest
COPE: Committee on Publication Ethics
DOI: Digital Object Identifier
ICMJE: International Committee of Medical Journal Editors
LMICs: Low- and Middle-Income Countries
PLOS: Public Library of Science
RW: Retraction Watch
WAME: World Association of Medical Editors
